# Prognostic role of the lymph node ratio in node positive colorectal cancer: a meta-analysis

**DOI:** 10.18632/oncotarget.12131

**Published:** 2016-09-20

**Authors:** Ming-Ran Zhang, Tian-Hang Xie, Jun-Lin Chi, Yuan Li, Lie Yang, Yong-Yang Yu, Xiao-Feng Sun, Zong-Guang Zhou

**Affiliations:** ^1^ Department of Gastrointestinal Surgery, West China Hospital, Sichuan University, Chengdu, China; ^2^ Department of Orthopedics, West China Hospital, Sichuan University, Chengdu, China; ^3^ Institute of Digestive Surgery and State Key Laboratory of Biotherapy, West China Hospital, Sichuan University, Chengdu, China; ^4^ Department of Oncology, Department of Clinical and Experiment Medicine, Linköping University, Linköping, Sweden

**Keywords:** colorectal cancer, lymph node, lymph node ratio, prognostic role, meta-analysis

## Abstract

The lymph node ratio (LNR) (i.e. the number of metastatic lymph nodes divided by the number of totally resected lymph nodes) has recently emerged as an important prognostic factor in colorectal cancer (CRC). However, the tumor node metastasis (TNM) staging system for colorectal cancer does not consider it as a prognostic parameter. Therefore, we conducted a meta-analysis to evaluate the prognostic role of the LNR in node positive CRC. A systematic search was performed in PubMed, Embase and the Cochrane Library for relevant studies up to November 2015. As a result, a total of 75,838 node positive patients in 33 studies were included in this meta-analysis. Higher LNR was significantly associated with shorter overall survival (OS) (HR = 1.91; 95% CI 1.71–2.14; *P* = 0.0000) and disease free survival (DFS) (HR = 2.75; 95% CI: 2.14–3.53; *P* = 0.0000). Subgroup analysis showed similar results. Based on these results, LNR was an independent predictor of survival in colorectal cancer patients and should be considered as a parameter in future oncologic staging systems.

## INTRODUCTION

Colorectal cancer (CRC) is the third most common cancer and the third leading cause of cancer death in the United States [1]. Lymph node status is accepted as one of the most important prognostic factors in colorectal cancer [2]. The classic staging system for colorectal cancer is the tumor node metastasis (TNM) staging system, which stages lymph node involvement according to the absolute number of positive lymph nodes [2]. However, the TNM system does not take into account examined tumor-free lymph nodes. Therefore, lymph node ratio (LNR) has recently emerged as an important prognostic factor and a suitable staging method for node positive patients [3–5]. Nevertheless, it was still under controversy due to contradictory LNR consequences in the previous studies [6, 7]. A previous systematic review considered the evidence on LNR as a prognostic factor in the colorectal cancer [3]. However, the main research tool for this study is systemic review (only four series submitted for meta analysis). Since many new studies in the last years have investigated this topic and the last review date was around ten year ago, we aimed to clarify the prognostic role of NLR in patients with lymph node-positive colorectal cancer and conduct the first meta-analysis on this topic.

## RESULTS

### Eligible and characteristics of studies

**Figure 1:**
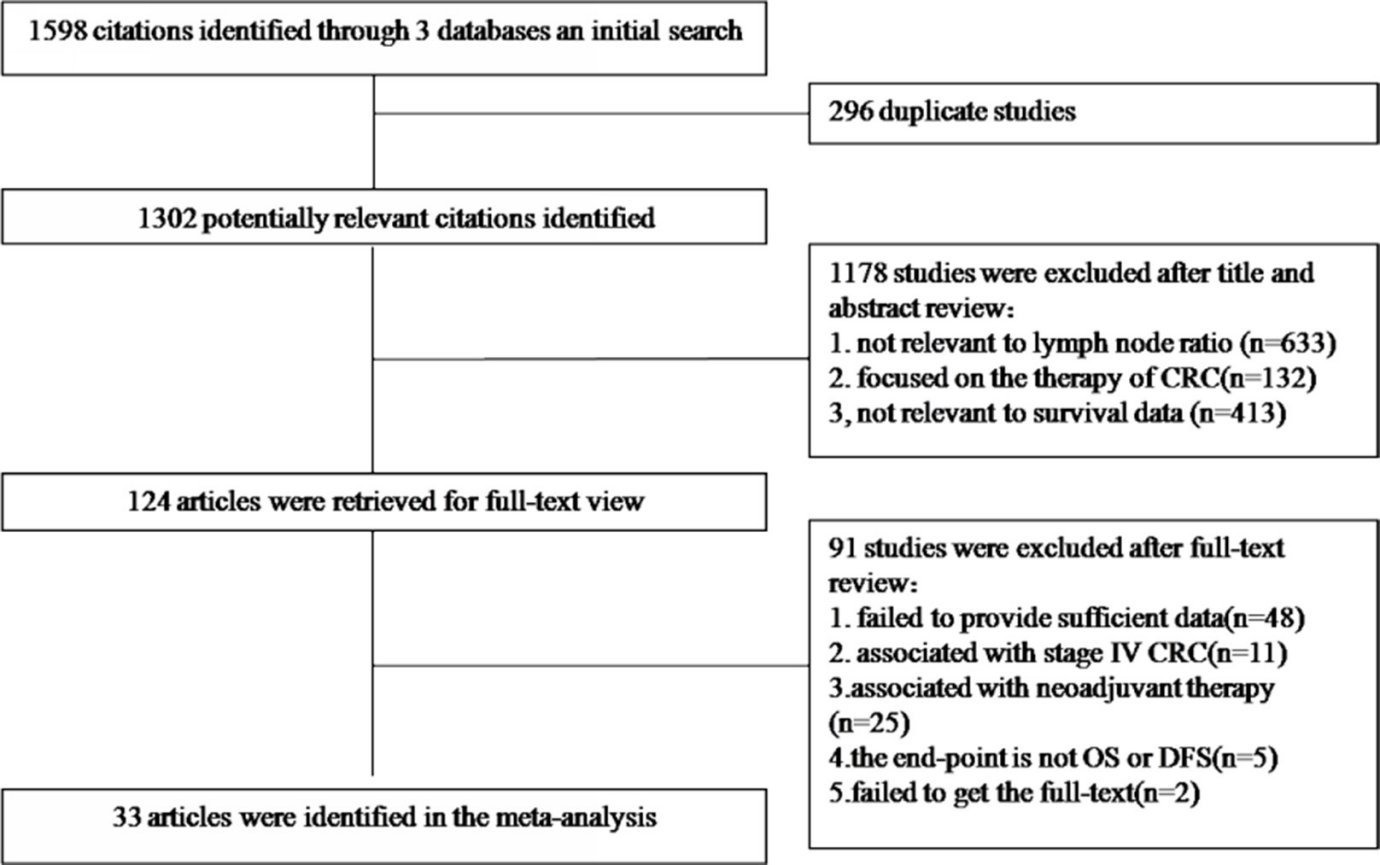
A flow chart showed the selection of studies

We identified 1598 potentially relevant articles from our search of the published literature. After removing duplications, scanning titles and abstracts and reading the full-text, 33 records [5, 7–38] encompassing a total of 81,331 (75,838 node positive) CRC patients were eligible for the present study based on our inclusion and exclusion criteria (Figure 1).

Demographic details and clinicopathologic characteristics of the included studies were summarized in Table 1 and Table 2. The 75838 node positive colorectal cancer patients were all underwent curative surgery, and their median age ranged from 54 to 75 years. Of all the 33 studies, 16 were focused on colon cancer, 5 on rectal cancer, and 12 considered both the colon and the rectum. We also investigated the situation of lymph nodes harvested and the treatment strategy (Table 2). The follow-up time ranged from 30.2 months to 86 months. The patients included in this study were diagnosed between 1991 and 2012.

**Table 1:** Demographic details of all identified studies

Study	Year	Sample	Patient age	Follow-up time	Country	Endpoint
Xue	2014	180	Median 54 years	Median 49 months	China	DFS
Arda	2014	58	Median 60 years	Mean 4-year	Turkey	OS DFS
Wang	2013	245	Median 61 years	Mean 6-year	China	OS
Yen	2013	612	Median 67 years	Median 52 months	Taiwan	OS, DFS
Tiago	2013	70	NA	Median 33 months	Brazil	DFS
Zhu	2012	161	Mean 59.1 years	NA	China	OS DFS
Liang	2012	174	Mean 62 years	Median 62.5 months	China	OS DFS
Kritsanasakul	2012	227	Mean 62.8 years	Median 86 months	Thailand	OS
Jung	2012	78	Median 64 years	Median 46 months	Korea	OS DFS
Shimomura	2011	266	Median 64 years	Median 42.4 months	Japan	DFS
Hong	2011	130	Mean 64 years	Median 50 months	Korea	DFS
Greenberg	2011	65	Mean 69 years	Mean 34 moths	Israel	OS,DFS
Vaccaro	2009	362	Mean 67.4 years	Median 42 months	Argentina	OS DFS
Galizia	2009	145	Median 66 years	Median 43 months	Italy	DFS
Wang	2012	256	Mean 57.9	Median 37 months	China	OS
Jing	2012	145	Median 66 years	Median 35.4 months	China	DFS
Tong	2011	505	Median 61 years	Median 31.08 months	China	OS
Shao	2011	282	NA	NA	China	OS
Jung	2010	514	Median 63 years	Median 48.5 months	Korea	OS DFS
Wang	2008	24477	Mean 69.2 years	NA	America	OS
Peng	2008	318	Mean 55.3 years	Median 41 months	China	OS, DFS
Derwinger	2008	265	Mean 72 years	Mean 3-year	Sweden	DFS
Lee	2007	201	Median 59 years	Median 41 months	Korea	DFS
Chin	2009	624	Mean 64.1 years	Mean 5-year	Taiwan	DFS
Arslan	2014	440	Median 66 years	Median 30.6 months	Turkey	OS
Kim	2009	232	NA	Median 53 months	Korea	OS
Kobayashi	2011	452	NA	Median 5.3 years	Japan	OS
Lykke	2013	3119	Median 72 years	Mean 5-year	Denmark	OS
Moug	2014	1514	Mean 71.9 years	Median 5.3 years	Scotland	OS
Thoma	2012	1908	Mean 68 years	Median 30.2 months	England	OS
Parnaby	2015	921	Median 75 years	Median 52.8 months	England	OS,DFS
Chen	2011	36712	Mean 69.6 years	NA	America	OS
Zhou	2015	180	Mean 59 years	Median 41.8 months	China	OS

“NA”: not available; “OS”: overall survival;”DFS”: disease free survival.

**Table 2:** Clinicopathologic characteristics of all studies

Study	Stage	Location	Inclusion period	Treatment	No. of nodes (N+)
Xue	III	colorectum	2007–2012	R0 surgery	median 8,(2)
Arda	III	colon	2006–2014	R0 surgery	NA
Wang	III	colorectum	2000–2006	R0 surgery + AT	NA
Yen	III	colorectum	2004–2008	R0 surgery + AT	median 18,(3)
Tiago	III	colon	2005–2010	R0 surgery	median 18.5
Zhu	III	rectum	2005–2010	R0 surgery	mean 13.4
Liang	III	colorectum	2000–2003	R0 surgery	median 10,(3)
Kritsanasakul	I–III	colorectum	1998–2007	R0 surgery + AT	median 10 (1.7)
Jung	I–III	colon	1999–2007	R0 surgery + AT	median 7
Shimomura	III	colorectum	1991–2008	R0 surgery + AT	median 14,(2)
Hong	III	colon	2000–2006	R0 surgery + AT	median 28,(2)
Greenberg	I–III	colorectum	2003–2009	R0 surgery + AT	median 16
Vaccaro	III	colorectum	1980–2005	R0 surgery + AT	median 20,(2)
Galizia	III	colon	1996–2007	R0 surgery + AT	median 15,(2)
Wang	III	colon	1999–2008	R0 surgery + AT	mean 23.3(4.2)
Jing	III	colon	1998–2008	R0 surgery + AT	mean 13.22(3.77)
Tong	III	colorectum	1994–2007	R0 surgery	median 12,(2)
Shao	II–III	colorectum	2000–2005	R0 surgery	mean 11.44(2.21)
Jung	III	colorectum	1998–2007	R0 surgery + AT	median 14,(2)
Wang	III	colon	1988–2003	curative surgery	NA
Peng	III	rectum	1990–2004	R0 surgery + AT	mean 12(3.8)
Derwinger	III	colon	1999–2003	R0 surgery + AT	median 11
Lee	III	colon	1995–2001	R0 surgery + AT	median 17,(3)
Chin	III	colon	1995–2003	R0 surgery + AT	NA
Arslan	I–III	colon	2005–2011	R0 surgery	median 19
Kim	III	rectum	1996–2006	R0 surgery + AT	median 17,(3)
Kobayashi	III	rectum	1991–1998	R0 surgery + AT	median 37(2)
Lykke	I–III	colon	2003–2008	R0 surgery	median 13(2)
Moug	I–III	colon	2000–2004	R0 surgery + AT	median 11
Thoma	III	colorectum	1997–2007	R0 surgery + AT	median 11(4)
Parnaby	I–III	colon	2006–2012	R0 surgery + AT	median 16
Chen	III	colon	1992–2004	R0 surgery	NA
Zhou	II–III	rectum	2005–2010	R0 surgery + AT	median 11(4)

“AT”: adjuvant treatment; “No. of nodes (N+)”: total number of lymph nodes harvested (number of positive lymph nodes); “NA”: not available.

All the HRs and their 95% CIs in the collected articles were listed in Table 3. We also summarized the methodological quality details. Firstly, the cut-off value of the LNRs was quite different from each other and stratified methods were not consistent (Table 3). Secondly, almost all of researchers used the multivariate statistical analysis models. Thirdly, most studies were retrospective study in design, while 5 articles were designed as the prospectively studies. Regarding the relationship between LNR and the clinicopathological characteristics of node positive colorectal cancer patients, no significant differences emerged for mean age and gender. Furthermore, the LNR was not associated with tumor location or T stage [15, 23, 39]. Higher LNR patients have, however, significant major proportion of a higher lymphovascular invasion and poor differentiation [15, 23, 39].

**Table 3:** Summary table of HRs (95% CI) and HR calculation

Study	HR (95%CI)	LNR cutoff value	LNR stratification	Statistical analysis	Study design
**OS**					
Arda	1.712 (0.982–2.984)	0.25	NA	MA	R
Wang	1.641 (1.099–2.450)	0.3	Log rank analysis	MA	R
Yen	1.54 (1.05–2.22)	0.17	Log rank analysis	MA	R
Zhu	3.655 (1.939–6.888)	0.43	Mean	MA	R
Liang	1.42 (1.13–1.76)	0.125, 0.26, 0.5	Quartiles	MA	R
Kritsanasakul	2.62 (1.79–3.85)	0.35, 0.69	ROC curve analysis	MA	R
Jung	1.402 (1.265–4.564)	0, 0.01, 0.28	Median value	MA	R
Greenberg	12.2 (2.178–68.622)	0.13	ROC curve analysis	MA	R
Vaccaro	2.3 (1.3–4.1)	0.25	Quartiles	MA	R
Wang	1.754 (1.344–2.289)	0.11, 0.39	Log rank analysis	MA	P
Tong	1.958 (1.652–2.321)	0.35, 0.69	Log rank analysis	MA	R
Shao	1.263 (1.027–1.552)	0, 0.17, 0.41, 0.69	Literature data	MA	R
Jung	1.589 (1.106–2.284)	0.18	Quartiles	MA	R
Wang	2.30 (2.083–2.545)	1/14, 0.25, 0.5	ROC curve analysis	MA	SEER
Peng	3.41 (1.63–7.13)	0.14, 0.49	Literature data	MA	R
Arslan	2.197 (1.357–3.556)	0.05, 0.20	NA	UA	P
Kim	2.261(1.234–4.143)	0.1, 0.2, 0.4	Quartiles	MA	R
Kobayashi	2.114 (1.241–3.600)	0.04, 0.079, 0.15	Quartiles	MA	R
Lykke	1.560 (1.232–1.975)	0, 1/12, 1/4, 1/2	Literature data	MA	P
Moug	2.117 1.350–3.318)	0.05, 0.19, 0.39	Literature data	MA	P
Thoma	1.799 (1.132–2.859)	0, 0.11, 0.21, 0.36, 0.60	NA	MA	P
Parnaby	2.464 (1.487–4.083)	0, 0.17, 0.41, 0.69	Literature data	MA	L
Chen	1.975 (1.519–2.568)	0.1, 0.24, 0.49, 0.99, 1	Log rank analysis	MA	SEER
Zhou	1.71 (1.1–2.65)	0, 0.19	ROC curve analysis	MA	R
**DFS**					
Xue	2.098 (1.050–4.192)	0.17	ROC curve analysis	MA	R
Arda	1.736 (0.997–3.024)	0.25	NA	MA	R
Yen	1.53 (1.05–2.23)	0.17	Log rank analysis	MA	R
Tiago	74.88 (1.55–3617.01)	0.15	Literature data	MA	R
Zhu	2.775 (1.544–4.988)	0.43	Mean	MA	R
Liang	1.39 (1.15–1.69)	0.125, 0.26, 0.5	Quartiles	MA	R
Jung	3.073 (1.496–6.313)	0, 0.01, 0.28	Median value	MA	R
Shimomura	2.425 (1.497–3.922)	0.2	ROC curve analysis	MA	R
Hong	5.868 (1.585–21.729)	0.1638	Quartiles	MA	R
Greenberg	3.297 (0.875–12.427)	0.13	ROC curve analysis	MA	R
Vaccaro	2.6 (1.5–4.8)	0.25	Quartiles	MA	R
Galizia	5.56 (3.45–12.5)	0.1818	ROC curve analysis	MA	R
Jing	11.75(3.20–43.12)	0.11, 0.20. 429	Quartiles	MA	R
Jung	1.596 (1.122–2.268)	0.18	Quartiles	MA	R
Peng	3.82 (1.96–7.47)	0.14, 0.49	Literature data	MA	R
Derwinger	10.6 (3.2–31.8)	0.12, 0.27, 0.4	Quartiles	MA	R
Lee	2.880 (1.950–4.253)	0.11, 0.24,	Quartiles	MA	R
Chin	3.915 (1.249–12.269)	0.4, 0.7	Log rank analysis	MA	R
Parnaby	2.877 (1.837–4.507)	0, 0.17, 0.41, 0.69	Literature data	MA	R

Study design is described as prospective (P) or retrospective (R). SEER surveillance, epidemiology, and end results cancer registry; L location cancer registry.

NA, not available; OS, overall survival; DFS, disease -free survival;

ROC curve: receiver operating characteristic curve. LNR: lymph node ratio,

MA, multivariate statistical analysis models; UA, univariate statistical analysis models.

### Meta-analysis results

**Figure 2:**
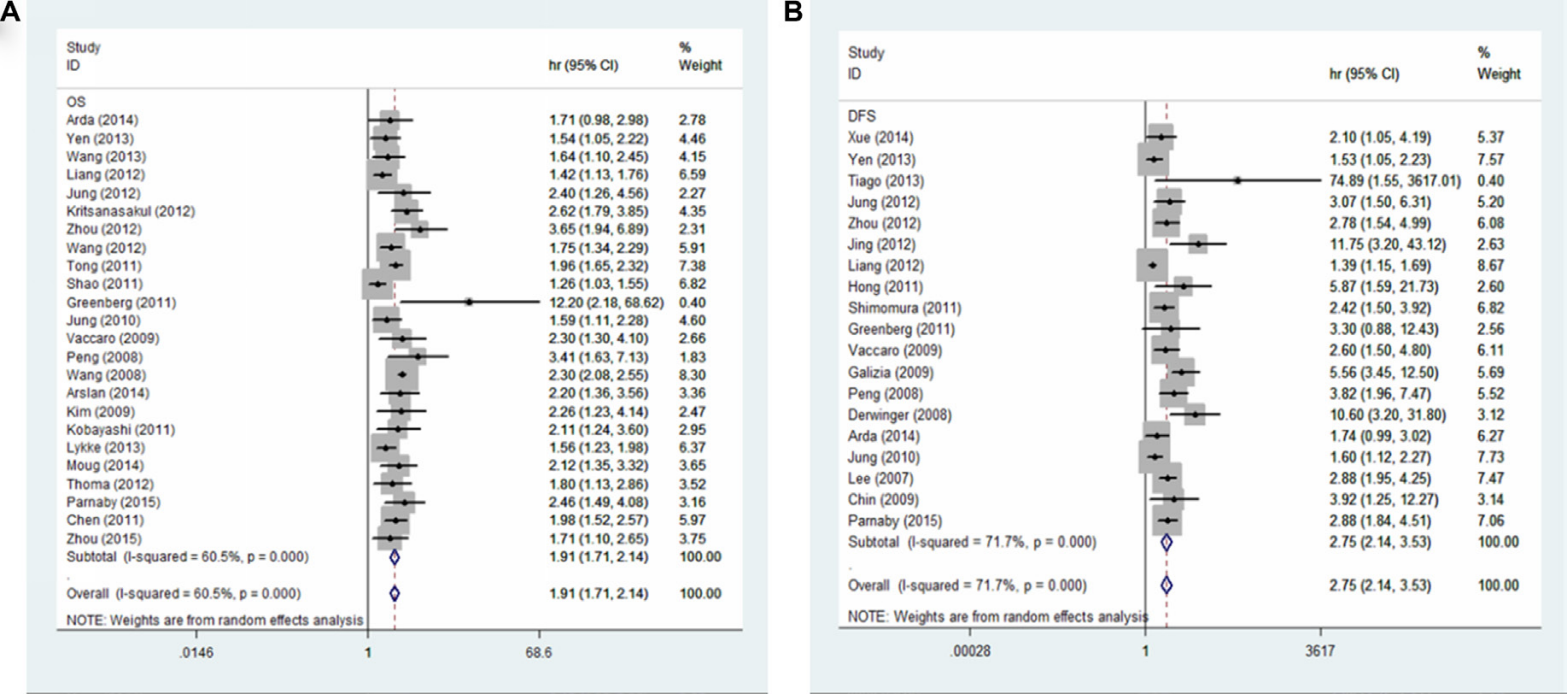
Forest plots show the association between LNR and overall survival (A), disease free survival (B)

As shown in Figure 2, a pooled HR and its 95%CI were calculated with a random model because of the heterogeneity test showed that statistically significant heterogeneity exists between the studies (for OS: *I*^2^ = 60.5%, *P* = 0.000; for DFS: *I*^2^ = 71.7%, *P* = 0.000). The result showed that elevated LNR may predict poor OS (*n* = 24) (the pooled HR was 1.91; 95% CI: 1.71–2.14) and DFS (the pooled HR was 2.75; 95% CI: 2.14–3.53). We next conducted subgroup analysis base on some important clinicopathological characteristics. The patients with higher LNR were all associated with decreased OS and DFS (Table 4).

**Table 4:** Results of the meta-analysis

Stratifications		No. of studies	Pooled Estimates		Model	Heterogeneity	
			HR (95% CI)	*P* value		*I*^2^(%)	*P* value
**OS**		24	1.91 (1.71–2.14)	0.000	R	60.5	0.000
**No. of nodes**	No. of nodes≥12	13	1.97 (1.71–2.26)	0.000	F	35.2	0.101
	No. of nodes<12	8	1.74 (1.40–2.17)	0.000	R	62	0.015
**Location**	Colon	9	2.11 (1.95–2.28)	0.000	F	35.1	0.137
	rectum	5	2.30 (1.79–2.96)	0.000	F	19.9	0.288
**Treatment**	R0 surgery +AT	15	1.96 (1.73–2.22)	0.000	F	8.8	0.355
	R0 surgery	9	1.83 (1.52–2.20)	0.000	R	81.3	0.000
**Stage**	Stage III	15	1.91 (1.71–2.14)	0.000	R	50.7	0.013
**DFS**		19	2.75 (2.14–3.53)	0.000	R	71.7	0.000
**No. of nodes**	No. of nodes≥12	13	2.87 (2.18–3.77)	0.000	F	48.8	0.062
	No. of nodes < 12	4	2.69 (1.32–5.50)	0.000	R	81.5	0.001
**Location**	Colon	9	3.49 (2.47–4.93)	0.000	R	48.9	0.048
**Treatment**	R0 surgery + AT	14	3.06 (2.32–4.04)	0.000	R	63.2	0.001
	R0 surgery	5	1.91 (1.27–2.86)	0.002	R	59	0.045
**Stage**	Stage III	16	2.73 (2.06–3.61)	0.000	R	74.6	0.000

“OS”: overall survival; “DFS”: disease free survival; “AT”: adjuvant treatment; “R”: random effects model; “F”: fixed effect model; “No. of nodes”: total number of lymph nodes harvested.

### Sensitivity analysis

**Figure 3:**
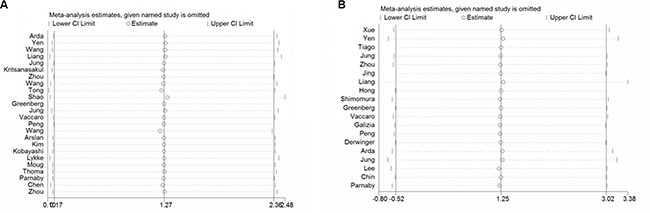
Sensitivity analysis of the association between LNR and overall survival (A), disease free survival (B)

Obvious heterogeneity was found in some analysis groups (Table 4). The most possible sources of heterogeneity were analyzed by subgroup. But subgroup analysis could not completely explain the heterogeneity. Therefore, we performed sensitivity analysis (Figure 3). In the OS analysis for all, heterogeneity was significant (*I*^2^ = 60.5%, *P* = 0.000). When Shaos’ study and Wangs’ study were removed from analysis, the heterogeneity became insignificant (*P* = 0.109 and *I*^2^ = 28.1%). As to DFS analysis for all (*I*^2^ = 71.7%, *P* = 0.000), we found that Liangs’, Yen's and Jungs’ study were responsible for the heterogeneity of DFS analysis group (*P* = 0.091 and *I*^2^ = 33.9%). After we excluded the publications with statistically significant heterogeneity and repeated the analysis, the summary estimates for higher LNR did not change statistically significantly (OS for all: the pooled HR was 1.85; 95% CI: 1.72–2.00; DFS for all: the pooled HR was 3.01; 95% CI: 2.55–3.55).

### Publication bias

**Figure 4:**
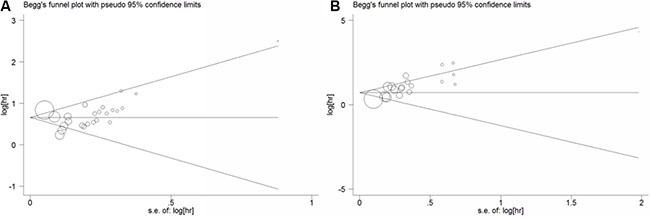
Funnel plot of the association between LNR and overall survival (A), disease free survival (B)

**Figure 5:**
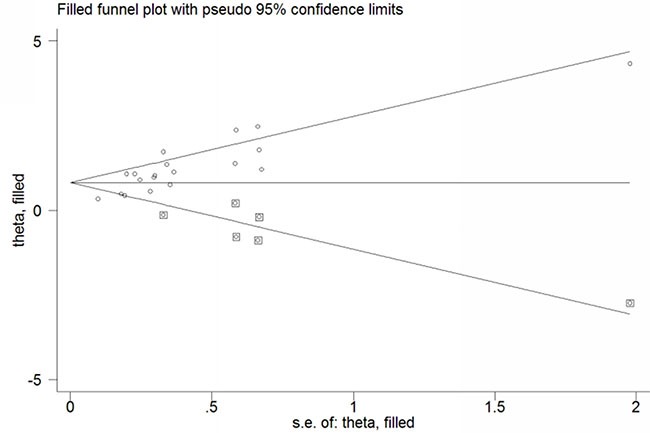
Trim and fill funnel plot for the source of publication bias

Funnel plots and Egger's test were conducted to evaluate the publication bias of included studies. No obvious visual asymmetry was observed in funnel plots (Figure 4) for OS, and the *P* values of the Egger's test were 0.800. However, statistically significant publication bias was found in the studies of DFS (Egger's test *P* value = 0.000). The funnel plot for the studies of DFS showed an asymmetrical distribution of the studies (Figure 4). Therefore we used the trim-and-fill method (Figure 5). As a consequence, there were 6 potential missing studies, and after these 6 potentially unpublished studies were filled, the recalculated pooled HR was 2.24 (95% CI: 1.75–2.88, *p* < 0.00001) in the random effects model. That indicated a positive outcome even though publication bias still exists.

## DISCUSSION

The prognosis of patients with colorectal cancer was largely related to the lymph node status, which helps in tumor staging and clinical decision. According to the current TNM staging system proposed by the AJCC/ UICC [2], N categories were determined by the absolute number of involved lymph nodes (N1, one to three; N2, four or more). Although this categorization has been proven to predict long term outcomes and well accepted [40], it is noteworthy that the TNM system does not take into account some important features of lymph node metastasis. In fact, many features of lymph node such as the number of non metastasis lymph nodes and the extra-nodal extension of nodal metastasis retrieved from the resection specimen which has been shown to have a prognostic significance in CRC [41, 42]. Furthermore, LNR can be considered as a hallmark of aggressiveness, since it was associated with a higher percentage of lymphovascular invasion and poor tumor differentiation [15, 23, 39].

In last decades, many researchers suggested that LNR could be a prognostic factor in different types of malignancies especially most of the gastrointestinal cancers [43–46]. This meta-analysis confirmed that higher LNR is statistically significantly associated with a poor survival of colorectal cancer. The results were similar when we subgroup the patients according to some important clinicopathological characteristics. Furthermore, we carried out a sensitivity analysis, which suggested the stability of our meta-analysis. We encountered evidence of publication bias in our main analysis, but our results remained unchanged after we adjusted for this. In current meta-analysis, we excepted the studies which included patients underwent neo-adjuvant treatment because it has reported that the total number of retrieved lymph nodes and positive lymph nodes may decrease after preoperative chemoradiation [47, 48].

Our results have demonstrated the significant weight of LNR in the prognosis of CRC. It is recommended to include LNR as a prognostic parameter in future colorectal staging system. It is important to note that the extent of dissection would influence the LNR. Generally, a more extensive surgical dissection of the specimen results in a higher number of positive nodes. And a ratio based on a small number of lymph nodes has a larger standard error, which could affect the reliability of the LNR in those patients who had less extensive dissection [49, 50]. So, adequate lymph nodes retrieved from the operative specimen was still important.

Our study had some advantages. First, this is the first complete meta-analysis identify the prognostic role of LNR in CRC. Second, this meta-analysis included plenty of primary studies (33 papers) and patients (75,838 node positive patients). The statistical power is well enough for our results. However, this study also had several limitations which are largely reflected by those within the primary studies. First, data about other co-morbidities (like cardiovascular diseases) were not reported, but it is known that they play an important prognostic role also in patients with cancer. Second, The cut-off value for defining LNR in each included study is quite different, which may have contributed to heterogeneity. Regarding which cutoff value will be the most reliable for predicting the prognostic values of colorectal cancer patients, the available evidence could not achieve an agreement. This needs a large cohort study or an individual patient data meta-analysis which could stratify and evaluate different LNRs on the CRC prognosis and find out the minute differences in prognostic outcomes. Finally, we also encountered some heterogeneity but were able to investigate sources of this within subgroup analysis and sensitive analysis.

In conclusion, this meta-analysis indicated that higher LNR can be used as a predictor of poor survival and assists in the choice of adjuvant treatment in the clinical setting in patients with CRC. We proposed that the LNR could be a prognostic parameter in future colorectal staging system.

## MATERIALS AND METHODS

### Search strategy and selection criteria

We systematically searched PubMed, Embase and the Cochrane library (http://www.cochrane.org) using the “lymph node ratio”, “LNR”;”lymph positive node ratio”, “lymph metastatic node ratio” Medical Subject Heading (MeSH) terms “Colorectal Neoplasms” and the individual corresponding free terms such as “colorectal cancer”, “colon cancer”, “rectal cancer” “colorectal adenocarcinoma”, “colon adenocarcinoma”, “rectal adenocarcinoma”, “colorectal carcinoma”, “colon carcinoma”, “rectal carcinoma”, “colorectal tumor”, “colon tumor”, “rectal tumor”. No language or other restrictions were applied. The last search was updated on 28 November, 2015. In addition, we reviewed references in the retrieved articles to search for additional relevant studies.

Studies eligible in the meta-analysis fulfilled the following inclusion criteria: (1) the patients were pathologically diagnosed as CRC with node-positive who underwent curative surgery (R0 resection);(2) the outcome of interest was overall survival (OS) and disease free survival (DFS);(3) hazard ratio (HR) and 95% confidence intervals (CI) were sufficiently reported. Exclusion criteria were defined as follows: (1) the patients have distant metastasis (TNM stage IV) or received neoadjuvant chemotherapy; (2) Letters, reviews, expert opinions, and case reports.

### Data extraction

The following information were extracted from each selected papers if available: first author, year of publication, country of the study population, number of patients, number of nodes examined, type of study, cut-off value for the LNR and definition of the strata, follow-up years, the location and the TNM stage of the tumor, and HRs with 95% CI. Two investigators reviewed and extracted information independently and checked by the other authors. Discrepancies were settled by consensus.

### Statistical analysis

The statistical analyses were carried out using STATA 12.0 (STATA Corporation, College Station, TX, USA). The HRs with 95% CI from each study were extracted to generate a pooled HR. Heterogeneity among studies was checked using the chi-squared test and I^2^ statistics. If the *P value* < 0.05 and/or I^2^ > 50% indicating statistical significance, a random effects model was used to obtain summary HRs. Otherwise, a fixed effect model was utilized. In addition, we conducted a sensitivity analysis to investigate the potential sources of heterogeneity and assess the strength of our findings by sequentially excluding one study. Furthermore, factors contributed to heterogeneities were also analyzed by stratifying the subjects according to the tumor location. Publication bias among the studies was investigated by using Begg's funnel plot and the Egger's test.
